# Macaque Area V2/V3 Reorganization Following Homonymous Retinal Lesions

**DOI:** 10.3389/fnins.2022.757091

**Published:** 2022-01-28

**Authors:** Georgios A. Keliris, Yibin Shao, Michael C. Schmid, Mark Augath, Nikos K. Logothetis, Stelios M. Smirnakis

**Affiliations:** ^1^Physiology of Cognitive Processes, Max-Planck Institute for Biological Cybernetics, Tübingen, Germany; ^2^Bio-Imaging Lab, Department of Biomedical Sciences, University of Antwerp, Antwerp, Belgium; ^3^Schmid Research Group, Medicine Section, University of Fribourg, Fribourg, Switzerland; ^4^Institute of Biomedical Engineering, ETH Zurich, Zurich, Switzerland; ^5^International Center for Primate Brain Research, Shanghai, China; ^6^Division of Imaging Science and Biomedical Engineering, University of Manchester, Manchester, United Kingdom; ^7^Department of Neurology, Brigham and Women’s Hospital and Jamaica Plain Veterans Administration Hospital, Harvard Medical School, Boston, MA, United States

**Keywords:** extrastriate cortex, fMRI, rhesus macaque, plasticity, visual cortex, reorganization

## Abstract

In the adult visual system, topographic reorganization of the primary visual cortex (V1) after retinal lesions has been extensively investigated. In contrast, the plasticity of higher order extrastriate areas following retinal lesions is less well studied. Here, we used fMRI to study reorganization of visual areas V2/V3 following the induction of permanent, binocular, homonymous retinal lesions in 4 adult macaque monkeys. We found that the great majority of voxels that did not show visual modulation on the day of the lesion in the V2/V3 lesion projection zone (LPZ) demonstrated significant visual modulations 2 weeks later, and the mean modulation strength remained approximately stable thereafter for the duration of our observations (4–5 months). The distribution of eccentricities of visually modulated voxels inside the V2/V3 LPZ spanned a wider range post-lesion than pre-lesion, suggesting that neurons inside the LPZ reorganize by receiving input either from the foveal or the peripheral border of the LPZ, depending on proximity. Overall, we conclude that area V2/V3 of adult rhesus macaques displays a significant capacity for topographic reorganization following retinal lesions markedly exceeding the corresponding capacity of area V1.

## Introduction

Understanding the detailed capacity of the adult visual system for plasticity is important as it may inform the design of rehabilitative treatments aiming to enhance visual recovery after injury.

Complete homonymous retinal injury eliminates the sensory input to retinotopically corresponding regions of visual cortex. Since the 1990s several studies have focused on the reorganization of the primary visual cortex (V1) after homonymous retinal lesions, producing in part conflicting results. Several studies reported substantial reorganization in the primary visual cortex of cats and monkeys following retinal lesions (Kaas et al., 1990; Heinen and Skavenski, 1991; Gilbert and Wiesel, 1992; Calford et al., 2000; Giannikopoulos, 2006; Gilbert and Li, 2012). Other reports, however, found minimal, if any, topographic changes (Murakami et al., 1997; Horton and Hocking, 1998; Smirnakis et al., 2005). In a recent review, Wandell and Smirnakis (2009) suggested that electrophysiological recording selection bias influences receptive field assessment, largely explaining the disparity of the results.

Similarly, several human fMRI case reports of patients with macular degeneration, suggest that human primary visual cortex undergoes large-scale reorganization (Baker, 2005; Baker et al., 2008; Schumacher et al., 2008; Dilks et al., 2009). Other studies, however (Sunness et al., 2004; Baseler et al., 2011), including a recent comprehensive report on 16 patients (Baseler et al., 2011), find no significant reorganization, or qualify the reported reorganization as being the result of task-dependent extra-retinal top-down feedback (Masuda et al., 2008; Haak et al., 2012; Barton and Brewer, 2015). For a comprehensive review on the capacity of V1 for reorganization following retinal lesions see Wandell and Smirnakis (2009). Overall, the weight of recent evidence (but see Gilbert and Li (2012) for a dissenting view) suggests that minimal, if any, feed-forward reorganization occurs in the primary visual cortex of cats or primates following retinal lesions. However, much less is known about the capacity of extrastriate cortex for reorganization following retinal lesions, and this is the question we tackle in this work.

Visual responsiveness of areas V2/V3 is often thought to depend entirely on V1 input. This is largely based on the finding that transient inactivation of area V1 by cooling immediately eliminates more than 95% of visually driven activity seen in retinotopically corresponding locations of areas V2/V3 (Schiller and Malpeli, 1977; Girard and Bullier, 1989; Girard et al., 1991). However, there is evidence that extrastriate cortex has the capacity to reorganize over longer time scales (weeks-months) following permanent area V1 aspiration lesions: a recent macaque study suggested that in the case of chronic V1 lesions visually driven BOLD responses can be elicited inside the lesion projection zone (LPZ) of areas V2 and V3 (Schmid et al., 2009). Furthermore, it was recently shown that this persistent activity as well as the monkeys’ residual visually based detection performance (“blindsight”), are mediated by inputs from the lateral geniculate nucleus (Schmid et al., 2010). An fMRI study in a human subject with hemianopia following area V1 injury also reports that visually driven activity persists in areas V2, V3, and V3A, arguing that this is likely the result of cortical reorganization (Baseler et al., 1999).

Although literature suggests that extrastriate cortex has the capacity for reorganization after V1 lesions, the question of what happens after retinal lesions remains open. It is possible that extrastriate cortex has different capacity for reorganization depending on the mechanism of deafferentiation (retina vs. V1). For one, subcortical pathways are thought to mediate the activity of areas V2 and V3 in the absence of V1 input in the case of V1 lesions (Schmid et al., 2010). Reports from the literature of “filling-in” studies suggest that in higher areas, under the right conditions, visual responses can be seen in cortical locations that are far away from the visual stimulus (Williams et al., 2008; Liu et al., 2010). For example, De Weerd et al. (1995) showed that the responses of extrastriate V2/V3 neurons whose receptive fields are contained inside an area devoid of visual stimulation (artificial scotoma) increase within seconds to reach a level comparable to that elicited by direct visual stimulation. Activation and perhaps enhancement of these pathways may be able to support reorganization.

In a recent case study on a juvenile macaque monkey with congenital dense bilateral macular degeneration (Shao et al., 2013), we reported that extensive reorganization was seen in area V5/MT. Here, we extend these results by using fMRI to study whether areas V2/V3 reorganize following bilateral homonymous retinal lesions induced by photocoagulation in adult macaques. The substantial topographic reorganization of V2/V3 demonstrated here is markedly different from previous findings in V1 of the same animals, which showed minimal, if any, reorganization following retinal lesions (Smirnakis et al., 2005). We argue that, in contrast to area V1, significant reorganization occurs in area V2/V3 under these conditions.

## Materials and Methods

### Subjects

Four healthy adult Macaca mulatta (M1, M2, M3, and M4) were used for these experiments. The data from these experiments have also been used in a previous study that examined reorganization in area V1 (Smirnakis et al., 2005). The experimental and surgical procedures were performed with care, in full compliance with the German Law for the Protection of Animals, the European Community guidelines for the care and use of laboratory animals (EUVS 86/609/EEC), and the recommendations of the Weatherall report for the use of non-human primates in research. The regional authorities (Regierungspräsidium Tübingen) approved our experimental protocol and the institutional representatives for animal protection supervised all procedures.

The monkeys were anesthetized during the fMRI experiments. Details on the anesthesia protocol have been given previously (Logothetis et al., 1999). Briefly, the animals were intubated after induction with fentanyl (31 μg/kg), thiopental (5 mg/kg) and succinylcholine chloride (3 mg/kg); anesthesia was maintained with isoflurane (M2 and M4) or remifentanyl (0.5–2 μg/kg/min, M1 and M3). Mivacurium chloride (5–7 mg/kg/h) was used after induction to ensure the suppression of eye movements. Heart rate and blood oxygen saturation were monitored continuously with a pulse-oxymeter. Body temperature was kept at 38–39°.

**FIGURE 1:**
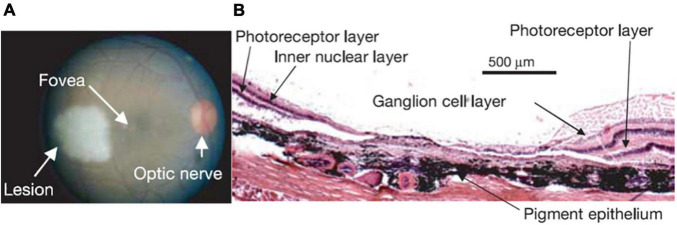
**(A)** Retinal lesion. The right retinal fundus 1–2 h following photocoagulation. The lesion appears pale white. Note that a corresponding lesion was made on the other side of the fovea in the left eye resulting in a homonymous left visual field scotoma (data not shown). Visual stimulation was always presented monocularly, on the right eye for this monkey, thus avoiding activity changes that may be due to potential eye misalignment. **(B)** Haematoxylin-eosin stain of a 15 μm thick section through the lesion in the right eye, where the visual stimulus was presented. Note the complete destruction of the photoreceptor layer, and the near complete destruction of the inner nuclear and ganglion cell layers (see Figure 1 in Smirnakis et al., 2005 for a full description).

Homonymous retinal lesions were induced by using a photocoagulation laser (NIDEKGYC-2000; 532 nm) under general anesthesia (Figure 1A) as described in Smirnakis et al. (2005). Histological results confirmed that the photoreceptor and bipolar cell layers as well as most of the ganglion cell layer of the extrafoveal retina were destroyed (Figure 1B). Histological confirmation was obtained in two of the four animals. Animal fundi were photographed in each experimental session, and we confirmed that all lesions remained stable throughout the course of the experiments (see Smirnakis et al., 2005 for the details).

### Stimuli

Stimuli were presented monocularly, at resolution of 640 × 480 pixels with a 60 Hz frame rate using an SVGA fiber-optic system (AVOTEC). Stimuli were centered on the fovea by using a modified fundus camera. Animals were fitted appropriate lenses to ensure the stimulus remained in focus. Standard expanding ring stimuli (outer radius expanded from either 0.3° or 0.9°–6.9° in steps of 0.6°, i.e., 12 or 11 annuli, frame interval = 6 s) and rotating wedge (90° wedges rotated in steps of 30°, i.e., 12 annuli, frame interval = 6 s) were presented to the subjects monocularly, always in the same eye for each animal. The full retinotopic maps were obtained pre-lesion but after lesioning only the eccentricity maps were followed to maximize repetitions and the signal to noise ratio of our measurements. The first measurement was obtained on the day of the lesion. The earliest subsequent measurement was obtained 14 days later to comply with the accepted animal protocol and allow for retinal recovery (Smirnakis et al., 2005).

One important control condition involved presenting the same expanding ring stimuli to the subjects and at the same time occluding a region in the normal half of the visual field. This region was designed to mirror the approximate location of the retinal lesion. We refer to this as the “artificial scotoma” (AS) condition. For all four subjects, the ASs were centered at (3.7°, 0) or (–3.7°, 0), on the opposite side of the scotoma resulting from the retinal lesion, and had a diameter of 3.7°.

FMRI experiments were performed on a 4.7T vertical scanner (Bruker Biospec, Bruker Biospin GmbH, Ettlingen, Germany). Multi-slice fMRI was performed by the use of 8 segmented gradient-recalled echo-planar imaging (EPI). The acquisition parameters were TE = 20 ms, TR = 750 or 805 ms, flip angle = 40°. Either 15 or 17 axial slices were collected at 1 × 1 mm^2^ in-plane resolution and 2-mm thickness. A full-brain anatomical scan was acquired before lesioning at 0.5 × 0.5 × 0.5 mm^3^ resolution for co-registration with the EPI images by using an MDEFT sequence (Logothetis et al., 1999; Keliris et al., 2007).

### Data Analysis

FMRI data were reconstructed and imported into a MATLAB based toolbox (mrVista)^1^ (Amano et al., 2009; Levin et al., 2010). The gray-white matter boundary was manually segmented using itkGray from the high resolution 3D-MDEFT anatomical images, and 3D cortical surface and flat mesh models were created and realigned with the functional data by using mrMesh/mrVista (Wandell et al., 2000).

In a typical experiment 5–10 repeats of the expanding ring and rotating wedge stimulation paradigm were performed and the average BOLD signal time course was generated. To obtain the retinotopic maps we have used the traveling wave method (Engel et al., 1997). The strength of the visual modulation was assessed using the measure of coherence, computed with the expanding ring stimuli. Coherence is defined as the BOLD signal spectral amplitude at the stimulus presentation frequency (12 or 11 cycles per scan in our experiments) divided by the average square root of the power over a range of nearby frequencies (Smirnakis et al., 2005). Normalized coherence was defined by dividing the coherence with the average coherence in a corresponding ROI in the contralateral (intact) hemisphere on the same day; this can account for variability across scanning days. The retinotopic maps were then fitted into a template of expected eccentricity and angle maps (atlas fitting) (Dougherty et al., 2003). During this procedure, the four sides of the visual field map, from fovea to periphery and from upper to lower vertical meridian were defined manually by simultaneously looking at angle and eccentricity gradients and were found to obey expected anatomical landmarks. For example, the dorsal V1/V2 border was confirmed to lay ∼2 mm from the lip of the lunate, the ventral V1/V2 border along the inferior occipital sulcus (Brewer et al., 2002), and the dorsal V2/V3 boundary at the bottom of the lunate sulcus. The calcarine sulcus was at ∼6.5° eccentricity as reported in Gattass et al. (2005). The fitting algorithm deforms these templates to match the eccentricity and angle data, allowing local deformations but no tears or folds in the atlas. The atlas with the least error compared with the data was generated, following (Dougherty et al., 2003). Visual inspection confirmed no major errors.

### Definition of the V2/V3 Lesion Projection Zone

**FIGURE 2:**
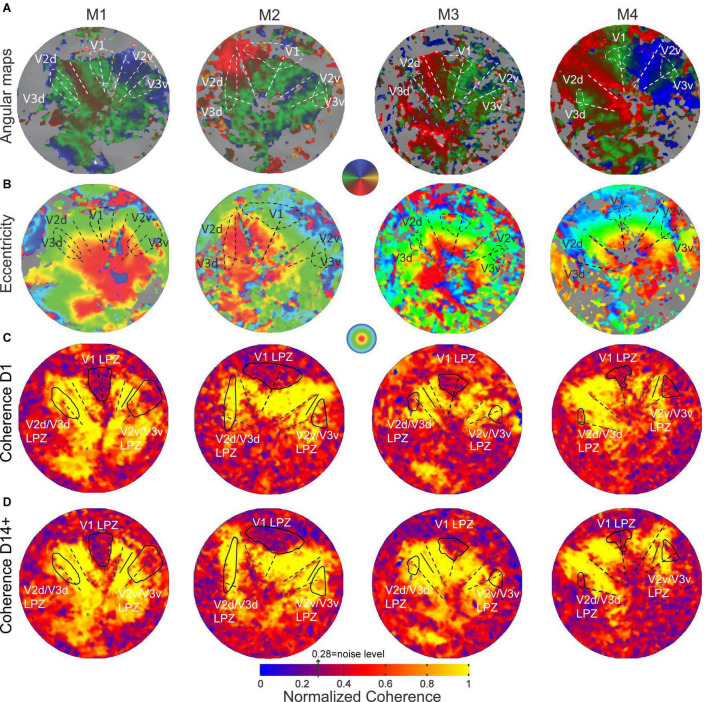
Pre-lesion retinotopy and LPZ definition. **(A)** Polar angle and **(B)** eccentricity functional activation maps obtained pre-lesion and overlaid on the flattened representation of early visual cortex in monkeys M1, M2, M3 and M4. **(C)** Normalized coherence maps of the hemispheres affected by the retinal lesion, obtained on the day of the lesion (D1) and **(D)** at least 14 days post-lesion using the ring stimulus (14 days post-lesion for monkeys M1, M3 and M4, 62 days post-lesion for M2). The V1 LPZs were selected directly based on the coherence maps (see section “Materials and Methods”). V2v/V3v and V2d/V3d LPZs were defined on the atlas fit of the pre-lesion retinotopic maps (Dougherty et al., 2003), by extracting voxels in areas V2 and V3 with similar eccentricities and polar angles as voxels in the V1 LPZ (see section “Materials and Methods”). The color map of coherence is normalized with respect to the average coherence across the voxels of an approximately iso-angular non-deafferented area V2 ROI (solid black line segment). The V1/V2 and V2/V3 borders and the V1 horizontal meridian are shown as dashed lines.

To find the V2/V3 LPZ we performed the following steps: (1) we selected V1 voxels with coherence below threshold. The value of 0.28 was chosen as a threshold based on the expected value of coherence in the absence of visual modulation that depends on the bandwidth (Δf = 12 cycles; thr = 1/(Δf + 1)^0.5^ = 0.28). This is a very conservative threshold for selecting the V1 LPZ as it equals the expected noise level ignoring its variability and thus selects the core of the V1 LPZ. Then, the voxels below threshold were merged to define the V1 LPZ (Figures 2C,D). Note that the area V1 LPZ has been shown to remain unchanged over time in this data set (Smirnakis et al., 2005), (2) we fitted the pre-lesion eccentricity and polar angle maps into an atlas per monkey using the process described in Dougherty et al. (2003), and (3) based on the pre-lesion retinotopic atlas-fit, the voxels in V2/V3 that corresponded retinotopically to the voxels belonging to the V1 LPZ, to within 0.05° of eccentricity and polar angle (on the atlas) were selected. These selected voxels were highly clustered and continuous inside the dorsal and ventral V2/V3. They were then merged to define the V2/V3 LPZ (Figures 2C,D). Once the V2/V3 LPZ was identified on the anatomical template, all quantitative analysis was done on the original data derived from voxels located inside the anatomical region corresponding to the LPZs. For each monkey, we followed the same procedure in order to define the artificial scotoma projection zones (ASPZ) in areas V1 and V2/V3 on the non-deafferented hemisphere (ipsilateral to the scotoma). Note that the monkeys were always scanned in the same position with the help of implanted fMRI-compatible headposts, and that the LPZ was defined on the high-resolution anatomical scan that serves as a template for aligning the functional data. These procedures assured we could follow the activity of the same anatomical region over time.

## Results

### Identification of the Lesion Projection Zones

Before lesioning the retina, retinotopic maps were measured using standard expanding rings and rotating wedge stimuli (see section “Materials and Methods”; Engel et al., 1997; Baseler et al., 1999). As shown in Figures 2A,B, all subjects showed normal retinotopic organization. The V1/V2 and V2/V3 borders were identified by the location of the vertical and horizontal meridians, respectively (Figure 2A). The border location was confirmed anatomically, as the macaque visual area boundaries have stereotypical anatomical locations (see section “Materials and Methods”).

Following a homonymous retinal lesion, parts of the visual cortex become deafferented (Heinen and Skavenski, 1991; Yinon et al., 1993; Smirnakis et al., 2005; Giannikopoulos, 2006). This results in a series of LPZs, one per retinotopic visual area. Identifying the LPZ of V1 is relatively straightforward given that, according to its role as primary visual cortex, V1 is receiving input from the lateral geniculate nucleus, it has relatively small receptive field sizes. In addition, a major part of central V1 which encompasses the LPZ in our study is exposed on the relatively flat operculum. On the contrary, identifying the LPZs in higher visual areas such as V2/V3 is more challenging given their anatomical location, receptive field sizes, and fewer numbers of voxels. Our goal was to define the LPZ in areas V2/V3 and follow how the strength of its visual modulation changed over time. Note that given that these areas share the horizontal meridian their LPZs are expected to be joined and dissected by the horizontal meridians and moreover to be positioned along the V2-V3 border both dorsally and ventrally. In order to determine a stable and appropriate LPZ for our V2/V3 analysis, we used the LPZ retinotopic coordinates carefully identified in V1 to guide our identification of the V2/V3 LPZ, instead of attempting to define the V2/V3 LPZ based on activity levels in the region which are expected to be variable over time. Importantly, because the areas V2/V3 have larger receptive fields in comparison to V1, this V2/V3 LPZ region is expected to be larger than the absolute V2/V3 LPZ. To this end, to be able to estimate the extent of this activity we have also used the stimuli that included an artificial scotoma in the healthy hemisphere and evaluated the timecourse of activity within the V2/V3 artificial scotoma projection zone (ASPZ) defined in an identical way as the V2/V3 LPZ.

### Visual Modulation Inside the V2/V3 Lesion Projection Zone

**FIGURE 3:**
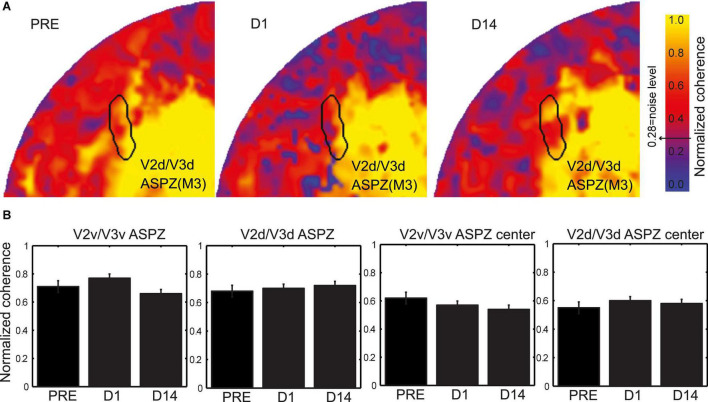
Coherence evaluation inside the artificial scotoma projection zone (ASPZ). **(A)** Normalized coherence maps of M3 V2d/V3d ASPZ are presented on the flattened healthy hemisphere. **(B)** Evaluation of the normalized coherence values across all subjects M1-M4 and time [Pre-lesion (PRE), lesion-day (D1), and fourteen days after lesion (D14)] in the ventral and dorsal V2/V3 ASPZ. The two panels on the left present data from the complete ASPZ while the two on the right data from the center of the ASPZ. Kruskal-Wallis tests revealed no significant differences of the normalized coherence over time in all cases.

The retinal lesion crosses the horizontal meridian and so the V2/V3 LPZ is split into two parts, one across the ventral and the other across the dorsal V2/V3 border (Figure 2C). From the coherence maps (Figure 2C) it is evident, as we expected, that even on the day of the lesion (D1), the V2/V3 LPZs are, on average, significantly visually modulated. Therefore the V2/V3 LPZ appears to receive visually modulated input outside the retinotopically corresponding V1 LPZ, which shows no significant visual modulation over its entire extent (Smirnakis et al., 2005). The average fraction of voxels inside the V2/V3 LPZ that were visually modulated (> 2 standard deviations above noise level; note that this is a conservative definition of modulated voxels inside an area that was defined based on V1 LPZ selected with a threshold at noise level) on the day of the lesion (D1) was 50.4 ± 12% (standard deviation across subjects). This was consistent with observations made in the artificial scotoma (AS) control condition: the average fraction of voxels that were visually modulated inside the ASPZ was similar at 53.5 ± 10% (standard deviation, Figure 3).

**FIGURE 4:**
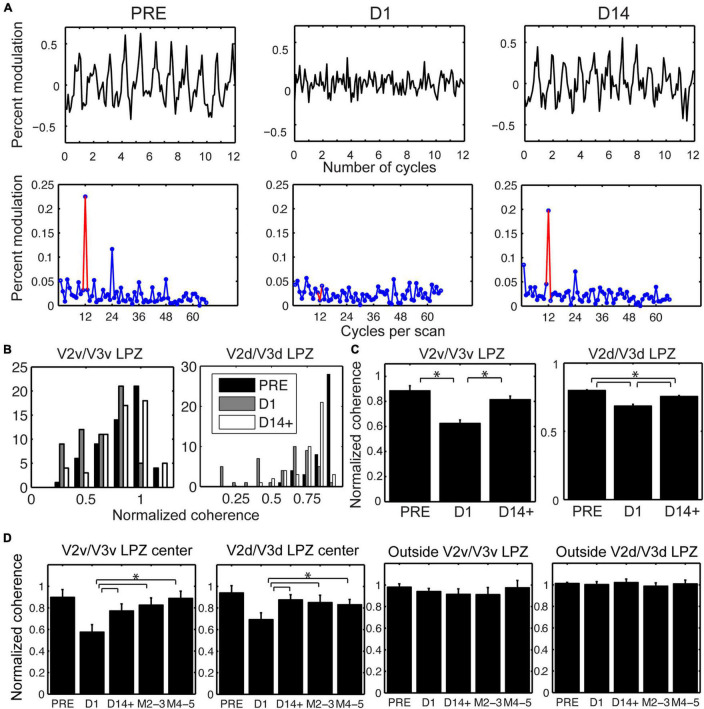
Longitudinal changes in the strength of visual modulation inside the V2/V3 LPZ. **(A)** Top row: Percent BOLD signal modulation as a function of stimulus cycle from voxels inside the V2v/V3v LPZ of M1 with low coherence (< 2 standard deviations above noise level on day 1), plotted pre-lesion (PRE) and on days 1 (D1) and 14 (D14) post-lesion. Bottom row: Average signal amplitude as a function of temporal stimulation frequency (12 cycles per scan, indicated by the red color). Note that the strength of the visual modulation recovers over time. **(B)** Distribution of normalized coherence values (see section “Materials and Methods”) for all functional voxels inside the V2v/V3v and V2d/V3d LPZs pre-lesion and at D1 and D14+ post-lesion histogrammed across all monkeys. Note that the number of voxels with low coherence inside the V2/V3 LPZ increases on the day of the lesion, then appears to substantially recover by D14+. **(C)** Mean normalized coherence across all functional voxels inside the V2v/V3v and V2d/V3d LPZ pre-lesion, as well as D1 and D14+ following the lesion, plotted across all monkeys. One–way ANOVA shows a significant effect for both LPZ’s over time (*F* = 76.48, *p* < 10^–8^ for V2v/V3v LPZ and *F* = 11.45, *p* < 10^–5^ for V2d/V3d LPZ). Tukey *post hoc* pairwise comparisons show that the strength of visual modulation dropped significantly on Day 1 compared to pre-lesion values, and then rose again significantly from Day 1 to D14+ post-lesion in both the ventral and the dorsal LPZ. Error bars represent s.e.m. *p < 0.05. **(D)** Mean normalized coherence across the voxels inside the V2v/V3v or V2d/V3d LPZ that were either within 2 standard deviations of noise level (LPZ center) or greater (Outside) on day 1 (lesion day; see section “Materials and Methods”) is plotted as a function of time across animals. Error bars represent s.e.m. *p < 0.05. Note that the mean coherence drops on the day of the lesion (D1) and then increases over time following the lesion (M2-3 and M4-5 refer to months after the lesion). This agrees with **(B,C)**, which represent the aggregate response across all animals.

Importantly, in addition to the voxels that were visually modulated immediately after the lesion, a fraction of voxels that were not significantly modulated on the day of the lesion (D1) recovered the ability to be visually modulated by day 14 post lesion (D14+, Figure 2D and Supplementary Figure 1). Figure 4A, top row, plots the percent modulation of the BOLD signal as a function of the stimulus cycle of voxels taken from monkey M1’s V2v/V3v LPZ with low coherence (< 2 standard deviations above noise level) on the day of lesion. Note how the strength of the visual modulation initially drops following the lesion (D1) but then recovers over time from day 1 (D1) to day 14 (D14). The same result is also demonstrated in Figure 4A, bottom row, which shows the signal amplitude as a function of temporal frequency for the same voxels and the modulations of spectral peak at the stimulus frequency (12 cycles/scan).

To better understand the reorganization of responses across days, we compared the distribution of coherence values measured across all the LPZ voxels pre-lesion (PRE) with the distribution on the day of the lesion (D1) and 14 days post-lesion (D14+) (Figure 4B). Because the overall level of the BOLD signal can fluctuate on different measuring sessions, the coherence of each voxel inside the LPZ was expressed as a fraction of the average coherence computed across all voxels within an iso-angular ROI (see Figures 2C,D) from a non-deafferented region of V2 in the same hemisphere (normalized coherence). Note that the distribution of normalized coherences derived from all voxels inside the V2/V3 LPZ (Figure 4B) shifts initially toward lower coherence values on the day of the lesion (D1) but returns close to pre-lesion levels 14 days later (D14+). One way ANOVA tests were performed comparing average normalized coherence over time (PRE, D1 and D14+) across all the V2v/V3v and V2d/V3d LPZ voxels in all 4 monkeys. There were significant differences of coherence over time in both the dorsal and ventral LPZ (*F* = 76.48, *p* < 10^–8^ for V2v/V3v LPZ and *F* = 11.45, *p* < 10^–5^ for V2d/V3d LPZ). Statistics were performed using the functional voxels (a total of 58, 45 voxels for the V2v/V3v and V2d/V3d LPZ, respectively, across all monkeys). Tukey *post hoc* pairwise comparisons showed significant differences between the mean level of coherence pre-lesion (PRE) and day 1 (D1), D1 and day 14 (D14+) for both LPZs, as well as the differences between PRE and D14+ for V2d/V3d LPZ (*p* < 0.05; Figure 4C). In fact, the number of voxels with low coherence inside the V2/V3 LPZ also changes following the lesion. The average fraction of voxels inside the V2/V3 LPZ that had low coherence (< 2 standard deviations above noise level) across subjects was 14.1 ± 8% (standard deviation) pre-lesion, increased to 49.6 ± 12% on D1, and dropped back to 14.8 ± 9% on D14+, close to pre-lesion levels.

To study how the strength of visual modulation inside the LPZ of area V2/V3 evolves over time, we focused on voxels in the center of the V2/V3 LPZ whose absolute coherence was low on day 1 (D1) following the lesion (< 2 standard deviations above noise level). For the sake of comparison, the coherence of each voxel in the LPZ was normalized by the average coherence across voxels of the non-deafferented control V2 ROI as described before (see Figure 2). As shown in Figure 4D, the mean coherence of these voxels was high pre-lesion, dropped on the lesion day, increased markedly as early as 2 weeks after the lesion and then remained approximately at the same level over time. In contrast, the same analysis in control voxels outside the V2/V3 LPZ did not show any significant changes in coherence levels (Figure 4D). Overall, for all four subjects, we found that the strength of the visual modulation inside the V2/V3 LPZ increased over time following the lesion, arguing for potential reorganization in area V2/V3. This contrasts with the case of the V1 LPZ where the signal dropped to noise levels following the lesion and did not change significantly over time (Smirnakis et al., 2005).

### Eccentricity Map Changes in V2/V3 Lesion Projection Zone

**FIGURE 5:**
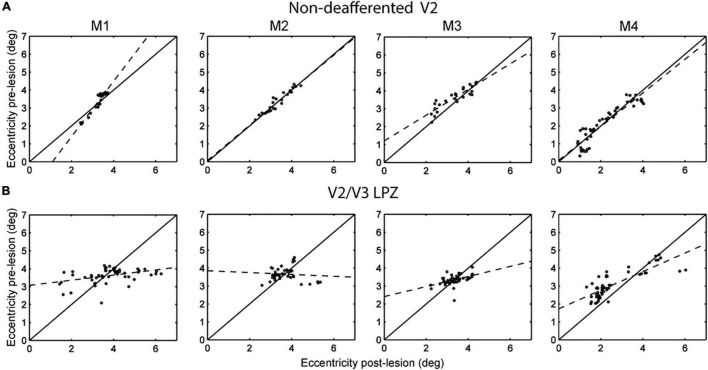
Eccentricity profile of V2/V3 LPZ. **(A)** Scatter plots of pre-lesion vs. post-lesion (day 14) eccentricities of voxels inside a control, non-deafferented, area V2 ROI (see Figure 2) near the V2/V3 LPZ border. Note that the eccentricities were mainly unchanged pre- and post- lesion (control). **(B)** Similar scatter plots of the voxels belonging in the dominant (largest) V2/V3 LPZ of each monkey. Note that eccentricities inside the LPZ span a wider range following the lesion, with eccentricities that started low tending to become lower and eccentricities that started higher tending to become higher. This suggests that lateral inputs are likely responsible for the activation of the V2/V3 LPZ region.

The next step was to look for the source of the input leading to the increase in visual modulation inside the V2/V3 LPZ over time. Although part of the sensory input of the voxels inside the LPZ has been cut off because of the retinal lesion, neurons inside the LPZ may still receive input from visual field locations that are outside the border of the scotoma (Barton and Brewer, 2015). To test this, we compared the range of eccentricities of the voxels inside the V2/V3 LPZ before and 14 days after the lesion. We focused on the voxels whose strength of visual modulation recovered as a function of time following the lesion. Such voxels had low coherence (< 2 standard deviations above noise) on D1, but were modulated above noise level both before and 14 days following the lesion. For each subject, we performed the eccentricity analysis in the dominant (largest) V2/V3 LPZ, dorsal or ventral. This was compared to controls (Figure 5A) from an approximately iso-angular non-deafferented area V2 ROI in each subject (see Figure 2). Eccentricity measurements in control ROIs (Figure 5A) demonstrate the reliability of this analysis. As expected, all control ROIs showed strong correlation between eccentricities measured pre-lesion and on day 14, with slopes near one (*b* = 1.54, 0.98, 0.71, 0.94; *R*^2^ = 0.91, 0.93, 0.79, 0.86; *p* < 10^–7^ for M1, M2, M3 and M4, respectively, illustrated in dashed lines). Figure 5B presents the eccentricity scatter plots of voxels inside the dominant V2/V3 LPZ before and after the lesion. Voxel eccentricities pre-lesion were significantly correlated with eccentricities measured on day 14 in 3 out of 4 subjects, but slopes were significantly smaller than one (*b* = 0.14, 0.28, 0.52; *R*^2^ = 0.17, 0.22, 0.67; *p* < 0.01 for M1, M3, and M4, respectively, and *b* = –0.05, *R*^2^ = 0.0084, *p* = 0.56 for M2; illustrated in dashed lines). Overall, the eccentricities inside the LPZ span a wider range following the lesion compared to pre-lesion for each subject. Voxels with small eccentricity pre-lesion tend to have smaller eccentricity after the lesion, while voxels with large eccentricity pre-lesion tend to have larger eccentricity after the lesion. This suggests that neurons inside the LPZ may regain their activity by receiving input either from the foveal or the peripheral border of the LPZ, depending on which one is closer.

## Discussion

This study analyzed the potential for reorganization in areas V2/V3 of the macaque following retinal lesions. In contrast to our previous findings in area V1 of the same animals that demonstrated no visually driven activity inside the V1 LPZ after the lesion (Smirnakis et al., 2005), here we found that the LPZs in areas V2/V3 could be visually modulated already at the first post-lesion imaging time point, 2–6 h following retinal photocoagulation. Given our derivation of the V2/V3 LPZs based on the V1 LPZ, we expected *a priori* to observe some visual modulations inside it given the larger receptive field sizes of areas V2/V3. However, we observed that approximately ∼50 ± 12% (standard deviation) of the voxels inside the V2/3 LPZ were visually modulated 2–6 h post-lesion. Notably, a similar fraction of visually modulated voxels was observed inside the V2/V3 projection zone of the artificial scotoma control condition (ASPZ). We conjecture that the surprisingly large extent of visual modulations we observed at this short time scale may indicate that additional factors may play a role. For example, our observations are consistent with De Weerd et al. (1995), who showed that multi-unit activity can be elicited in area V2 and V3 but not area V1 locations, if distant surround is visually stimulated. A dynamic change in the balance between excitation and inhibition affecting the range over which receptive fields receive inputs may be the cause of this relatively rapid adaptation (Knierim and van Essen, 1992). Given its rapid time course, this phenomenon is likely to be mediated by pre-existing wiring, although we cannot exclude conclusively the possibility that plasticity mechanisms operating over the course of several hours may play a role.

Our results show that, over longer time periods, V2/V3 has a noticeable capacity for reorganization. Overall, the great majority of the V2/V3 LPZ voxels that do not show visual modulation on the day of the lesion (D1) can be visually modulated 2 weeks later. The mean coherence of V2/V3 LPZ voxels whose activity drops hours after the lesion (D1) increases markedly by 2 weeks post-lesion and remains approximately at the same level thereafter (see Figures 4C,D). In marked contrast, visually driven activity does not recover inside the area V1 LPZ, whose border remains stable to within 1 mm (Smirnakis et al., 2005). Therefore, the increase in the strength of visual modulation inside the V2/V3 LPZ is unlikely to be caused by area V1 recovery or even by significant retinal surround recovery, which presumably would first leave a signature in area V1.

Figure 5B suggests that following the retinal lesion, the eccentricities of visually modulated voxels inside the V2/V3 LPZ span a wider range than pre-lesion. The form of the eccentricity change suggests that neurons inside the LPZ may reorganize by receiving intra-areal input either from the foveal or the peripheral border of the LPZ, depending on which one is closer. It is likely that this plastic reorganization is mediated by local cortico-cortical connections between the V2/V3 LPZ and its surround. Strengthening of projections from the surround of the V1 LPZ to area V2 may also play a role. We believe that similar processes likely operate along iso-angular lines. However, since we followed reorganization just by using expanding/contracting ring stimuli, we cannot definitively prove this.

Subcortical projections from the surround of the retinal lesion itself are not likely to play a major role (although that possibility cannot be completely ruled out), as they would first be expected to influence the extent of the V1 LPZ, which does not change. Moreover, this is supported by prior work by Schmid et al. (2010) who studied area V2 activity within the LPZ induced by area V1 lesions. Specifically, Schmid et al. (2010) showed that the activity that returns within the LPZ in V2 following area V1 lesions is mediated primarily by inputs from the LGN, since lidocaine infusions in LGN completely abolished visual modulation in the V2 LPZ. In this case inputs from the retina to the SC and Pulvinar to V2 are presumably intact but they do not seem to contribute. Although in our case we induced retinal rather than V1-lesions (retinal lesions would impair at least part of the projection to SC and pulvinar), we suspect that this reasoning still applies making it unlikely that the Pulvinar or the SC are the source of the recovery of visual modulation inside the V2 LPZ. One might wonder whether filling-in of the LGN LPZ from the intact retina surrounding the lesion could play a role, but we consider this also to be unlikely because in this case we would have expected the V1 LPZ to exhibit significant filling-in. Trans-callosal connections between retinotopically corresponding locations across hemispheres are also unlikely to play a major role as: (1) one would expect the range of eccentricities represented inside the LPZ to approximately match the pre-lesion values, which is not the case (Figure 5B), and (2) in some of the experiments an artificial scotoma was presented on the side contralateral to the actual scotoma, depriving the contralateral cortex from retinotopically corresponding visual input. Scenarios involving higher areas and pathways involving feedback are also unlikely as the monkeys were under anesthesia during the experiments.

To address potential effects of anesthesia influencing the experimental findings, care was taken for the conditions of anesthesia to be stable across the experimental time points. Moreover, the stability of the border of visual evoked activity in area V1 across time provides an additional validation of the stability of anesthesia and indicates that any variability in the conditions of anesthesia was not able to modulate cortical activity differentially across time points (Smirnakis et al., 2005). On the other hand, there is a possibility that the effect of anesthesia might underestimate the degree of the return of visually driven modulation we describe.

The recovery from retinal stunning due to photocoagulation in not likely to contribute to the activity in the V2/V3 LPZ we measured at D14+. Were the retinal recovery from stunning to be a major contribution to the recovery seen, one would have expected to see a change in the border of the LPZ in area V1 also, since this is the first relay of information from retina to the cortex (through LGN). Since this was not observed (Smirnakis et al., 2005), it is unlikely that retinal recovery can be the cause of the observed recovery in area V2.

It is interesting to speculate on the difference between V2/V3 reorganization following a retinal lesion vs. following an area V1 lesion (Schmid et al., 2009). In the retinal case, activity inside V2/V3 LPZ returns to nearly normal levels (Figure 4C). By contrast Schmid et al. (2009) showed that visual modulation strength in area V2/V3 following a V1 lesion returns to only ∼20–30% of prior activity levels and depends mostly on subcortical projections from the LGN (Schmid et al., 2010). This difference is surprising in view of the fact that retinotopically corresponding parts of area V2/V3 were visually deprived in both cases, and V2/V3 LPZ size was commensurate in both studies. This suggests that the existence of healthy V1 cortex and its upstream connections may be important for potentiating part of the reorganization seen in extrastriate areas after retinal lesions. Another possibility is that the surgery to lesion area V1 (Schmid et al., 2009, study) might have injured some of the subcortical en-passant fibers targeting V2, thereby limiting the potential recovery in this case. Alternatively, the existence of functional retinotopically corresponding subcortical inputs, which are preserved in the case of the V1 lesion but silenced in the case of the retinal lesion, may limit the ability of lateral intra-areal connections to reorganize.

Human subjects suffering from macular degeneration exhibit perceptual filling-in in their blind visual field as well as an associated distortion of visual space (Gerrits and Timmerman, 1969; Kapadia et al., 1994; Burke, 1999; Zur and Ullman, 2003). It has been argued that primary visual cortex is able to reorganize (Darian-Smith and Gilbert, 1994; Darian-Smith and Gilbert, 1995; Calford et al., 2003) and could provide a contribution to this phenomenon (Gilbert and Li, 2012). However, the degree of V1 reorganization following retinal lesions has been questioned (Murakami et al., 1997; Horton and Hocking, 1998; Smirnakis et al., 2005; Wandell and Smirnakis, 2009), and De Weerd et al.’s (1995) filling-in study in animals with an intact visual system suggests that higher areas may be involved. Our results suggest that adult extrastriate cortex exhibits significant capacity for reorganization following retinal injury, and may potentially contribute to the phenomenon of perceptual “filling-in” in subjects with retinal lesions.

## Data Availability Statement

The raw data supporting the conclusions of this article will be made available by the authors, without undue reservation.

## Ethics Statement

The animal study was reviewed and approved by the Regierungspräsidium Tübingen.

## Author Contributions

GK supervised, analyzed the data, and wrote the manuscript. YS analyzed the data. MS performed the experiments and edited the manuscript. MA provided support in experiments. NL designed the study, provided infrastructure support, and edited the manuscript. SS designed the study, performed experiments, supervised, and wrote the manuscript. All authors contributed to the article and approved the submitted version.

## Conflict of Interest

The authors declare that the research was conducted in the absence of any commercial or financial relationships that could be construed as a potential conflict of interest.

## Publisher’s Note

All claims expressed in this article are solely those of the authors and do not necessarily represent those of their affiliated organizations, or those of the publisher, the editors and the reviewers. Any product that may be evaluated in this article, or claim that may be made by its manufacturer, is not guaranteed or endorsed by the publisher.

## Footnotes

1
http://white.stanford.edu/software/
