# Supplementary material for: Macaque Area V2/V3 Reorganization Following Homonymous Retinal Lesions
Source: Front Neurosci. 2022 Jan 28;16:757091. doi: 10.3389/fnins.2022.757091 (PMC8832035; doi:10.3389/fnins.2022.757091)
Supplement: Supplementary file 1 [file Image_1.pdf]

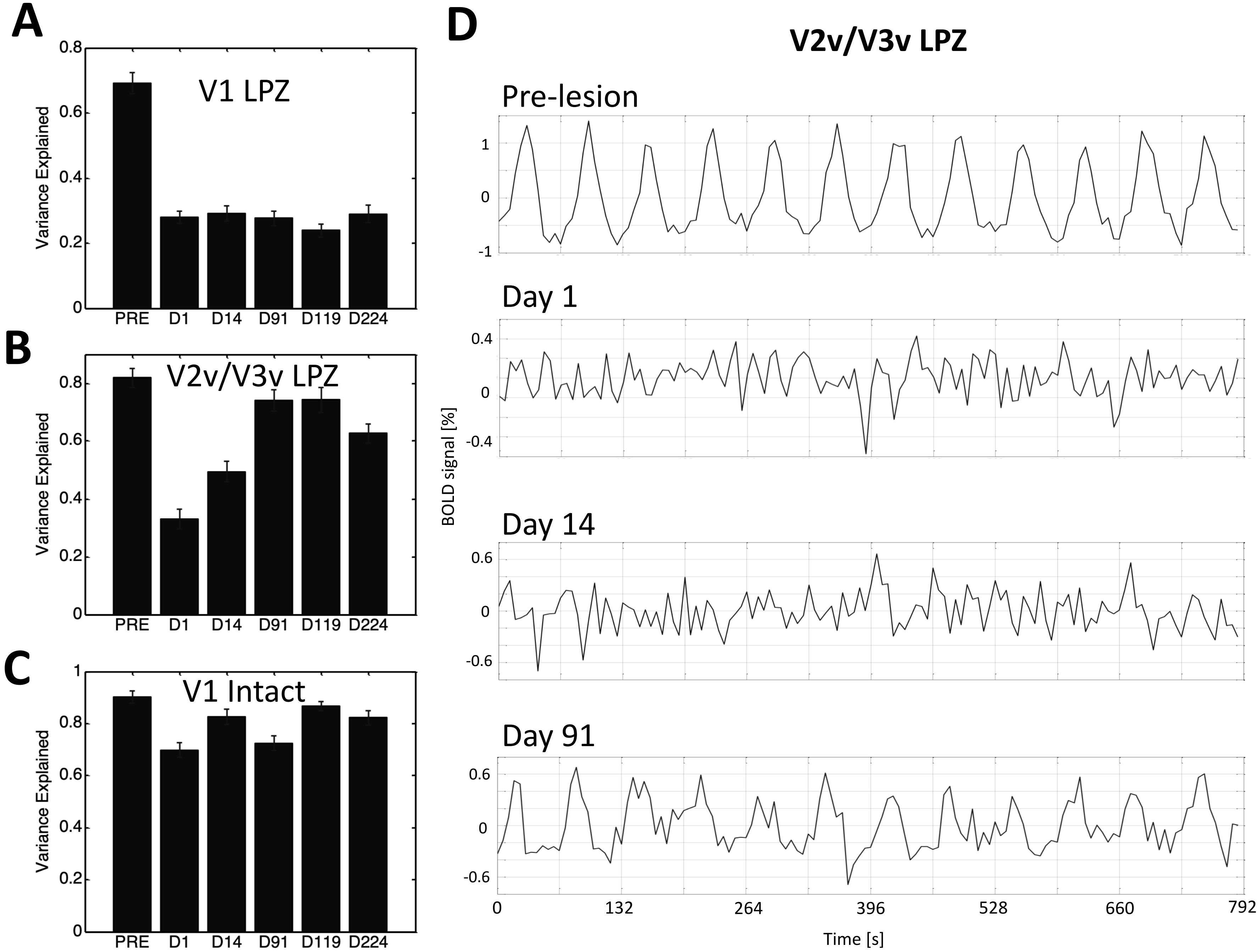

Supplementary Figure 1: Longitudinal changes in the strength of visual modulation in V2/V3 LPZ in comparison to V1 lesion projection (LPZ) and intact zones. **(A)** Fraction of variance explained by the model predictor representing the visual stimulation paradigm before the lesion (PRE) and different timepoints in number of days after the lesion (D1, D14, D91, D119, D224). **(B)** The same longitudinal change in fraction of explained variance as (A) but in V2v/V3v LPZ of the same subject. **(C)** The same change in fraction of explained variance as (A,B) but in the intact V1 zone. **(D)** Time-courses of percent BOLD signal changes induced by visual stimulation (12 ON-OFF cycles / scan) in selected time-points in the V2v/V3v LPZ. Note the gradual recovery of visual modulation after the initial drop following the lesion.
